# Continuous use of direct oral anticoagulants during and after simple and surgical tooth extractions: a prospective clinical cohort study

**DOI:** 10.1186/s12903-025-05949-9

**Published:** 2025-04-12

**Authors:** Krister Johansson, Jonas P Becktor, Aron Naimi-Akbar, Peter J Svensson, Bengt Götrick

**Affiliations:** 1 Department of Oral and Maxillofacial Surgery and Oral Medicine, Faculty of Odontology, Malmö University, Malmö, Sweden; 2 Department of Oral & Maxillofacial Surgery, Skåne University Hospital, Lund, Sweden; 3 Health Technology Assessment-Odontology (HTA-O), Faculty of Odontology, Malmö University, Malmö, Sweden; 4 Department of Translational Medicine, Clinical Coagulation Research Unit, Skåne University Hospital, Malmö, Sweden; 5 Department of Orofacial medicine, Faculty of Odontology, Malmö University, Malmö, Sweden

**Keywords:** Anticoagulants, Factor Xa inhibitors, Direct oral anticoagulants, Warfarin, Oral haemorrhage, Oral surgical procedures

## Abstract

**Background:**

No consistent approach to the management of direct oral anticoagulants (DOACs) during and after oral surgery has been established. Thus, DOACs may be unnecessarily discontinued, raising the potential risk of life-threatening thromboembolism. To address the inconsistency in this approach, our study assessed the risk of bleeding and other complications in patients who continue to use DOACs during and after simple and surgical tooth extractions.

**Methods:**

Between May 2016 and December 2023, this prospective study recruited patients aged 18 years or older who were receiving a DOAC or warfarin and were in need of simple or surgical extractions of one or more teeth. Local haemostatic agents were being used to control bleeding. Patients were instructed to manage minor postoperative bleeding at home by biting down on gauze soaked in tranexamic acid for at least 30 min. After surgery, all patients were followed for 7 days. The chi-squared test compared dichotomous variables; the two-sample *t*-test, continuous variables; logistic regressions, dichotomous outcomes; and linear regressions, continuous outcomes.

**Results:**

In all, 354 teeth were extracted from 160 patients receiving DOACs and 56 patients receiving warfarin. The incidence of any type of postoperative bleeding was 27% in patients receiving DOACs and 37% in those receiving warfarin (OR 0.66, 95% CI: 0.28–1.57; *p* = 0.35). Most patients were able to manage any bleeding at home themselves. Clinically relevant bleeding necessitating prompt evaluation or a secondary surgical intervention by a dentist or healthcare professional occurred in 3% of patients receiving DOACs and 11% of patients receiving warfarin (OR 0.30, 95% CI: 0.08–1.06; *p* = 0.06). No reports of major bleeding requiring hospitalization or blood transfusion were found. Perioperative bleeding volume was comparable between the two groups.

**Conclusions:**

Patients receiving DOACs without interruption during surgery may have a lower risk of bleeding than those on warfarin. Patients may safely continue to use DOACs during and after simple and surgical extractions. This eliminates the potentially higher risk of serious thromboembolic events that are associated with a pause in anticoagulant therapy.

**Clinical trial registration:**

ClinicalTrials.gov (ID: NCT04662515). Retrospectively registered 4 December 2020.

**Supplementary Information:**

The online version contains supplementary material available at 10.1186/s12903-025-05949-9.

## Introduction

The growing prevalence of patients receiving direct oral anticoagulants (DOACs) presents a challenge for general dental practitioners when planning surgical procedures. The most frequent oral surgical procedure that dentists face is tooth extraction, which may require temporary discontinuation of anticoagulants to reduce the risk of bleeding. However, guidelines for managing patients receiving DOACs during simple and surgical tooth extractions are inconsistent [1, 2]. This inconsistency may lead to unnecessary discontinuation of DOACs, which can increase the potential risk of life-threatening thromboembolism [3]. Therefore, clear guidelines for managing DOACs in general dental practice during minor oral surgical procedures are needed.

For several reasons, use of DOACs has been on the rise compared to warfarin. DOACs have been demonstrated to be as effective as warfarin in preventing stroke and are associated with a reduced risk of bleeding, particularly intracranial bleeding. Other advantages of DOACs include minimal drug and food interactions and a wide therapeutic range that allows for standardised dosing. Furthermore, DOACs do not require frequent monitoring, resulting in significantly fewer blood tests for patients [4].

A recent systematic review suggests that patients receiving DOACs may have a lower risk of bleeding after tooth removal than those receiving warfarin. Nevertheless, the quality of the evidence was low or very low due to the relatively small number of patients in the studies [5]. In contrast, this clinical study hypothesized that patients receiving DOACs without interruption have a higher risk of bleeding after minor oral surgery procedures compared to those receiving warfarin and have an international normalised ratio (INR) of ≤ 3.1. The objective of this study was to assess the risk of bleeding and other complications in patients who continue to use DOACs during and after simple and surgical tooth extractions.

## Materials and methods

### Study design

This prospective clinical cohort study examined complications, including peri- and postoperative bleeding, in patients who underwent one or more simple or surgical tooth extractions and were receiving either a DOAC (DOAC group) or warfarin (warfarin group).

### Study population and setting

Between May 2016 and December 2023, patients referred to three Swedish oral surgery departments were queried concerning enrolment in the study: the Department of Oral and Maxillofacial Surgery and Oral Medicine in the Faculty of Odontology at Malmö University, the Department of Oral and Maxillofacial Surgery at Skåne University Hospital, and the Department of Oral Surgery at the Public Dental Service in Skåne, Lund. The study included patients aged 18 years or older who required one or more simple or surgical tooth extractions and were taking a DOAC without interruption and had an estimated glomerular filtration rate (eGFR) > 30 mL/min/1.73 m^2^ or were taking warfarin and had an INR ≤ 3.1. INR was measured preoperatively on the same day or the day before surgery. Patients taking both antiplatelets and anticoagulants were instructed to stop their antiplatelet medication from 7 days before the surgical intervention until 7 days after. Exclusion criteria comprised non-adherence to prescribed anticoagulant medication, previous radiation therapy exceeding 30 Gy at the surgical area, ongoing alcohol or drug abuse, or previous participation in the present study. To prevent selection bias, all eligible patients who fulfilled the inclusion and exclusion criteria and who had been referred to a participating department were invited to participate.

### Clinical procedures

The use of a case report form ensured that the treatments were performed in a standardised manner. Eligible patients were clinically examined and their medical history was retrieved. Immediately before the surgical procedure, venous blood samples were collected to determine haemoglobin values, and systolic and diastolic blood pressure readings were measured. Local anaesthesia (Xylocaine^®^ adrenaline 20 mg/mL + 12.5 µg/mL, Dentsply DeTrey GmbH) was administered peripherally and/or as regional blocks. This study includes all teeth that needed extraction, regardless of their anatomical position. In each patient, one to eight teeth were extracted. Extractions were classified as simple or surgical. Simple extractions involved luxation, elevation, separation if necessary, and the use of forceps. With or without bone removal, if a flap was raised, the extraction was classified as surgical. Any granulation tissue was removed. Two patients underwent apical surgery on an adjacent tooth. A local resorbable haemostatic agent (Surgicel^™^, Ethicon US, LLC, Spongostan^™^ Dental, Ethicon US, LLC or Lyostypt^®^, B. Braun Surgical) was frequently placed in the socket. Resorbable sutures were used, and complete wound closure was done after most surgical extractions. Following the procedure, patients were instructed to bite down on a roll of gauze soaked in a tranexamic acid solution (100 mg/mL) for at least 30 min. In the event of any subsequent bleeding, patients were instructed to repeat the procedure. Additionally, patients were advised to rinse with chlorhexidine 0.1% twice a day for 7 days after the procedure. For pain relief, patients were instructed to take paracetamol at a dose of 4 g per day (2 g per day for patients taking warfarin) and to use oxycodone 5 mg as needed. Patients were followed up by telephone or at the clinic 7–28 days after surgery, depending on their general health. Any patient who could not be reached within 28 days were contacted later. All postoperative complications requiring treatment, and all bleeding episodes were recorded. If bleeding could not be managed at home, patients were instructed to contact the clinic for assistance.

### Outcome variables

The primary outcome measure was postoperative bleeding. All instances of bleeding that occurred after the patient’s departure from the clinic and necessitated compression or more comprehensive interventions were recorded, irrespective of whether they were managed by the patient or healthcare personnel. Bleeding was classified according to the Bleeding Academic Research Consortium as grade 1–5 [6]. Briefly, grade 1 includes bleeding that patients can manage at home; grade 2, clinically relevant bleeding that requires prompt evaluation or a second surgical intervention from a dentist or healthcare professional; and grades 3–5, more severe types of bleeding.

Secondary outcomes comprised perioperative bleeding volume, the surgeon’s assessment of the impact of bleeding on the procedure’s complexity, and all postoperative complications requiring treatment by a dentist or other healthcare professional. All postoperative outcomes were calculated based on data from the first 7 postoperative days.

Perioperative blood loss was calculated by multiplying the total fluid volume in the suction bottle by its haemoglobin value, and then dividing the product by the haemoglobin value of a venous blood sample taken directly before surgery. The volume of liquid collected in the suction bottle was determined by weighing the filled bottle postoperatively and subtracting the weight of the unfilled bottle. It was assumed that 1 g of liquid equals 1 mL. The balances used for measuring had a readability of 0.01 g and a linearity of 0.05 g and were calibrated in accordance with the manufacturer’s instructions. Before surgery 5,000 units of heparin were added to the aspirate bottle to prevent blood clotting. Johansson et al. describes the blood collecting procedure, assay methods, and equipment in detail [7].

The surgeon assessed the impact of bleeding on the complexity of the procedure using a visual analogue scale (VAS), where 0 corresponds to no impact and 10, a substantial impact.

### Statistics

The sample size was calculated based on the incidence of grade 2 postoperative bleeding in patients on warfarin of 4% [8], and assuming a 16% incidence in patients receiving DOACs. A sample size calculation with type 1 error set to 0.05 and power set to 80% showed that 94 patients would be required in each group.

A patient cannot be included in an analysis if any of the variables needed for the analysis are missing. Thus, when possible, missing data were replaced in order to maximise the number of patients eligible for the analysis. Three patients receiving DOACs had minimal bleeding, resulting in haemoglobin values below the limits of detection of the analyses we used [7]. For those patients, bleeding was set to the lowest volume of perioperative bleeding recorded in the study before any analyses were done. Before regression analyses were done, other instances of missing data were also replaced. For one patient who had been prescribed warfarin, the length of the surgical procedure was not noted in the records. The missing value was assigned the median duration of surgical procedures for extracting the same number of teeth using the same surgical technique. Two patients receiving DOACs and two receiving warfarin did not have their blood pressure recorded. The missing values were assigned the median values for systolic and diastolic blood pressure in the data set.

To determine any differences between DOAC and warfarin users, we used the chi-squared test to compare dichotomous variables and two-sample *t*-tests to compare continuous variables. The study used logistic regressions to compare dichotomous outcome measures and linear regressions, continuous outcomes. The variables included in each regression model were selected based on their relative quality as determined by the Akaike information criterion [9], as well as their clinical relevance for each outcome. All models were adjusted for tooth removal from the upper jaw as a dichotomous variable and diastolic blood pressure as a continuous variable. Except for the model describing complications other than postoperative bleeding, all models were adjusted for dichotomous variables, including surgical extraction and the removal of multiple teeth. Furthermore, three outcomes (any type of postoperative bleeding, perioperative bleeding, and the impact of bleeding on the complexity of the procedure) were adjusted for (1) systolic blood pressure as a continuous variable, (2) diabetes, gender, and extraction of molars as dichotomous variables, (3) the departments where the treatments were performed and age as dichotomous indicator variables with three categories, and (4) country of birth and level of education as dichotomous indicator variables with four categories. Finally, two of the outcomes, perioperative bleeding and impact of bleeding on the complexity of the procedure, were also adjusted for the volume of local anaesthetic administered per tooth as a continuous variable.

All subgroups were analysed using the same models presented in the additional material (Additional file 1), except for the model that examined the association between INR and any type of postoperative bleeding. This model was adjusted for the continuous variables of systolic and diastolic blood pressure as well as the dichotomous variables of gender, diabetes, tooth removal from the upper jaw, surgical extraction, removal of molars, and removal of multiple teeth.

All analyses were conducted using STATA 18.0 SE. The threshold for statistical significance was set to a *p*-value of < 0.05, an odds ratio (OR) with a 95% confidence interval (CI) that did not include 1, and a regression coefficient with a 95% CI that did not include 0.

## Results

### Patient characteristics and interventions

No significant differences were observed regarding age, gender, or type of anticoagulant between the patients who underwent surgery (*n* = 216) and those who were not included in the study (*n* = 87). In all, 354 teeth were extracted from the 210 patients who constituted the final study population. Of the patients, 154 received a DOAC and 56, warfarin. Except for the characteristics discussed below, no significant differences were observed between the DOAC and the warfarin groups for any patient or procedural characteristic listed in Tables 1 and 2. The proportion of patients with atrial fibrillation was significantly higher in the DOAC group (*p* = 0.01; Table 1). Warfarin was administered with significantly greater frequency to patients with artificial valves (*p* < 0.01; Table 1). The proportion of patients who received local haemostatic agents in all extraction sockets was significantly higher in the warfarin group (*p* = 0.03; Table 2). Differences between the study groups concerning the identity of the surgeon, the department to which the patients were referred, or the department in which they were treated were not significant.

No patients were lost to follow-up. Two patients receiving DOACs continuously took a lower dose than recommended by their general practitioner. Analyses of the outcomes with and without these two patients resulted in no significant changes in any of the outcomes.

**Fig. 1:**
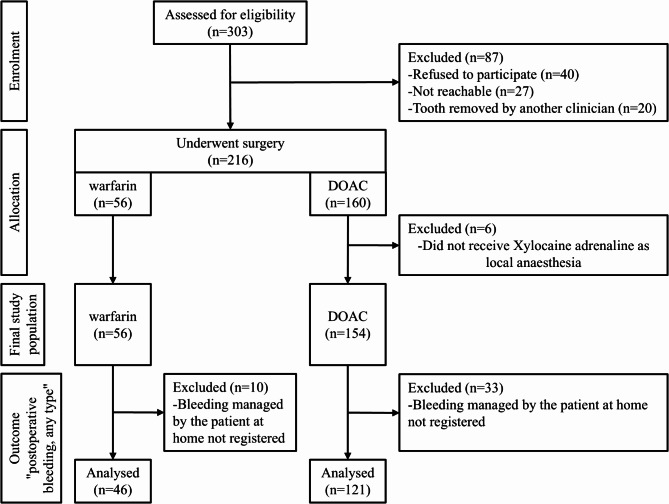
Study flow chart

Patients with missing data were excluded from some of the analyses, depending on the type of missing data. When analysing postoperative bleeding in general (all types), we excluded 33 patients in the DOAC group and 10 in the warfarin group. When analysing perioperative bleeding, we excluded 3 patients from each group. When analysing the impact of bleeding on the complexity of the procedure, we excluded 1 patient from the warfarin group. Figure 1 presents a flow chart of the study.

**Table 1:** Patient characteristics

Characteristic		Anticoagulant	
		DOAC (*n* = 154)	Warfarin (*n* = 56)
Age, median (IQR)		76 (69–81)	76 (64–81)
Age, n (%)			
	below 65 65–79 80 and above	22 (14) 81 (52) 51 (33)	14 (25) 25 (45) 17 (30)
Female, n (%)		75 (49)	19 (34)
Level of education, n (%)			
	lower secondary or lower upper secondary post-secondary or higher unknown	52 (34) 41 (27) 52 (34) 9 (6)	22 (39) 16 (29) 11 (20) 7 (13)
Country of birth, n (%)			
	Sweden Europe, except for Sweden non-European unknown	117 (76) 17 (11) 6 (4) 14 (9)	37 (66) 7 (13) 4 (7) 8 (14)
Diabetes mellitus type I and II, n (%)		30 (19)	14 (25)
Daily smoker, n (%)		11 (7)	1 (2)
Atrial fibrillation, n (%)		118 (77)	33 (59)
Venous thromboembolism, n (%)		24 (16)	9 (16)
Pulmonary embolism, n (%)		21 (14)	8 (14)
Artificial heart valve, n (%) type		3 (2) biological	15 (27) mechanical
Systolic blood pressure (mmHg), median (IQR)		140 (129–156)	136 (124–155)
Systolic blood pressure, n (%)			
	140 mmHg and below above 140 mmHg	84 (53) 76 (48)	31 (55) 25 (45)
Diastolic blood pressure (mmHg), median (IQR)		83 (77–92)	82 (70–93)
Haemoglobin level (g/dl), median (IQR)		13.7 (12.6–14.6)	13.7 (12.7–15.0)
eGFR, median (IQR) [minimum, maximum]		63 (53–75) [31, 110]	
DOAC type, n (%)			
	dabigatran rivaroxaban apixaban edoxaban	16 (10) 34 (22) 100 (65) 4 (3)	
DOAC dosing regimen*, n (%)			
	higher lower	115 (75) 39 (25)	
INR, median (IQR) [minimum, maximum]			2.4 (2.1–2.7) [1.2, 3.1]
INR, n (%)			
	Below 2.0 2.0–2.5 Above 2.5		9 (16) 27 (48) 20 (36)

DOAC: Direct oral anticoagulants, IQR: interquartile range, mmHg: millimetres of mercury, eGFR: estimated glomerular filtration rate, INR: International normalised ratio

*DOAC dosing regimen: Higher dose = dabigatran 150 mg twice a day, rivaroxaban 20 mg daily, apixaban 5 mg twice a day, edoxaban 60 mg daily. Lower dose = dabigatran 110 mg twice a day; rivaroxaban 15 mg daily, 10 mg daily; apixaban 2.5 mg twice a day; edoxaban 30 mg daily. Lower dose includes dosing out of regimen: dabigatran 150 mg daily, apixaban 2.5 mg daily

**Table 2:** Procedural characteristics

Characteristic		Anticoagulant	
		DOAC (*n* = 154)	Warfarin (*n* = 56)
Number of teeth extracted, median (IQR) [maximum]		1 (1–2) [7]	1 (1–2) [8]
Extraction of ˃ one tooth, n (%)		45 (29)	22 (39)
Extraction site (jaw), n (%)			
	upper only lower only both	56 (36) 83 (54) 15 (10)	28 (50) 24 (42) 4 (7)
Tooth extracted (type), n (%)			
	Molar Premolar Incisor or canine	108 (70) 40 (26) 34 (22)	39 (70) 17 (30) 15 (27)
Extraction indication, n (%)			
	Impaction Caries Pathological resorption Chronic apical periodontitis Radicular cyst Chronic periodontitis Retained dental root	2 (1) 21 (14) 5 (3) 51 (33) 0 23 (15) 72 (47)	2 (4) 13 (23) 2 (4) 22 (39) 1 (2) 7 (13) 22 (39)
Root under bridge, n (%)		8 (5)	5 (9)
Local anaesthesia (mL/tooth), median (IQR)		5.4 (3.6–7.2)	5.1 (2.7–5.4)
Extraction method, n (%)			
	simple only surgical only both	39 (25) 107 (69) 8 (5)	15 (27) 37 (66) 4 (7)
Removal of bone, n (%)		97 (63)	39 (70)
Apical surgery of adjacent tooth, n (%)		1 (1)	1 (2)
Primary closure rate, n (%)		105 (68)	37 (66)
Local haemostatic agent in all sockets, n (%)		120 (78)	51 (91)
Compression with tranexamic acid postoperatively, n (%)		149 (97)	54 (96)
Duration of surgery (minutes), median (IQR)		23 (16–31)	25 (17–35)
Duration of surgery < 24 min, n (%)		82 (53)	25 (45)
Start of surgery at 12 noon or later, n (%)		70 (45)	29 (53)

DOAC: Direct oral anticoagulants, IQR: interquartile range

### Outcomes

In the study cohort, 30% experienced postoperative bleeding of some kind within the first 7 postoperative days. All postoperative bleeding was classified as grade 1 or 2. Consequently, no instances of extensive postoperative bleeding requiring blood transfusion or hospitalization, or resulting in death, were observed. Most patients were able to manage their bleeding at home. The unadjusted regression analysis of grade 2 bleeding revealed a significant difference between the groups (OR 0.28, 95% CI: 0.08–0.96), but in the adjusted regression analysis, no significant difference was observed (OR 0.30, 95% CI: 0.08–1.06). The incidence of grade 2 bleeding necessitating additional sutures was 2.6% (4 of 154 patients) in the DOAC group and 7.1% (4 of 56) in the warfarin group. Nevertheless, no between-group difference was significant (unadjusted OR 0.35, 95% CI: 0.08–1.44; adjusted OR 0.40, 95% CI: 0.09–1.72). In relation to postoperative infections, perioperative bleeding volume, or the surgeon’s assessment of the impact of bleeding on the complexity of the procedure, no between-group difference was significant. Except for bleeding, the only postoperative complication that required intervention within the first 7 days was local infection (5% of the patients). All infections were treated with systemic antibiotics. Table 3 presents details of the outcomes.

**Table 3:** Associations between the anticoagulant groups and the outcome measures

Outcome	*n* (%)	Unadjusted values			Adjusted values	
		Odds ratio (95% CI)	*p*		Odds ratio (95% CI)	*p*
*Postoperative bleeding*,* any type*						
Warfarin (*n* = 46)	17 (37)	1			1	
DOAC (*n* = 121)	33 (27)	0.64 (0.31–1.31)	0.23		0.66 (0.28–1.57)	0.35
*Postoperative bleeding*,* Grade 2*						
Warfarin (*n* = 56)	6 (11)	1			1	
DOAC (*n* = 154)	5 (3)	0.28 (0.08–0.96)	0.04		0.30 (0.08–1.06)	0.06
*Other complications* ^*a*^						
Warfarin (*n* = 56)	2 (4)	1			1	
DOAC (*n* = 154)	8 (5)	1.48 (0.31–7.19)	0.63		1.18 (0.23–5.91)	0.84
	**Median (IQR)**	**Coefficient (95% CI)**	***p***		**Coefficient (95% CI)**	***p***
*Perioperative bleeding volume (mL)*						
Warfarin (*n* = 53)	6.27 (2.58–9.86)	0			0	
DOAC (*n* = 151)	4.92 (2.19–9.58)	0.39 (-3.07–3.84)	0.83		0.36 (-3.02–3.75)	0.83
*Impact of bleeding on procedure complexity* ^*b*^						
Warfarin (*n* = 55)	0 (0–0.60)	0			0	
DOAC (*n* = 154)	0.10 (0–0.90)	0.10 (-0.18–0.38)	0.47		0.16 (-0.12–0.45)	0.27

Cl: confidence interval, DOAC: direct oral anticoagulants, IQR: interquartile range

^a^ Other than postoperative bleeding

^b^ Assessment of the surgeon

### Subgroups

In the warfarin group, the INR level was associated with a risk of postoperative bleeding. The risk of any type of postoperative bleeding was significantly higher in patients with an INR of 2.6–3.1 compared with those with an INR of 2.0–2.5 (unadjusted OR 6.00, 95% CI: 1.46–24.73; adjusted OR 8.50, 95% CI: 1.48–48.89). Two patients in the warfarin group required cessation of anticoagulant therapy. The first, a 75-year-old female with a preoperative INR of 3.0 underwent surgical removal of tooth 28 (FDI World Dental Federation notation) and concomitant apical surgery on the buccal roots of teeth 26. Five days postoperatively, she developed bleeding with an INR of 6.8. The bleeding was controlled with compression, warfarin was discontinued, and she received vitamin K. A potential cause of the elevated INR was high paracetamol intake. The second patient, a 73-year-old male, had a preoperative INR of 2.4 and underwent surgical removal of tooth 37. Three days postoperatively, the patient developed local infection and bleeding. Systemic antibiotics were prescribed, and warfarin was discontinued. Additional suturing and debridement were performed seven days postoperatively. Two other patients in the warfarin group had a preoperative INR of 3.1. Both experienced postoperative bleeding with one patient presenting with grade 2 bleeding.

A detailed analysis was conducted on the DOAC group. No significant differences were observed between patients with an eGFR in the range of 31–44 mL/min/1.73 m^2^ and those with an eGFR ≥ 45 mL/min/1.73 m^2^. No instances of grade 2 bleeding or postoperative infections were reported for patients with an eGFR in the range of 31–44 mL/min/1.73 m^2^. Patients receiving higher doses of DOACs were then compared with those receiving lower doses (see explanations in Table 1). Although surgeons reported bleeding to have a greater effect on procedure complexity in the higher-dose group (OR 0.38, 95% CI: 0.05–0.71), the difference was not significant after adjustment (OR 0.29, 95% CI: -0.10–0.68). The lower-dose group reported no instances of grade 2 bleeding. No significant differences in the outcome measures were observed concerning the various DOACs prescribed.

An analysis of the group for whom perioperative bleeding was recorded (*n* = 204) was done to determine whether perioperative bleeding predicted any other outcome. The unadjusted and adjusted analyses revealed that each millilitre of increased perioperative bleeding was accompanied by a 0.05-point increase in the VAS score for the impact of bleeding on procedure complexity (95% CI: 0.04–0.06).

### Other variables

Additional file 1 presents the complete output of the regression models. The risk of postoperative bleeding was significantly higher in cases where more than one tooth was extracted, where surgical duration was prolonged, and in patients with higher systolic blood pressure. Patients with a systolic blood pressure of 170 mmHg displayed a twofold increase in the probability of any type of postoperative bleeding, from 20 to 40%, compared to patients with a systolic blood pressure of 110 mmHg.

## Discussion

The primary objective of this clinical study was to evaluate the risk of postoperative bleeding in patients who continued to use DOACs during and after simple and surgical tooth extractions. No instances of extensive postoperative bleeding requiring blood transfusion or hospitalization, or resulting in death, were observed in either the DOAC or the warfarin groups. The incidence of clinically relevant bleeding necessitating prompt evaluation or a secondary surgical intervention by a dentist or healthcare professional was lower in the DOAC group (OR 0.30, 95% CI: 0.08–1.06).

The reasons for some of the inclusion and exclusion criteria need to be clarified. Patients on DOACs with severe renal impairment (eGFR < 31 mL/min/1.73 m2) were excluded. This was because of the potential for increased risk of bleeding due to platelet dysfunction that may occur as a result of severe kidney disease. Additionally, these patients are at risk of DOAC accumulation, as DOACs are eliminated to varying degrees by the kidneys. Furthermore, one of the DOACs is contraindicated in this group [2, 10]. Patients on warfarin with an INR > 3.1 were also excluded. This threshold corresponds well with the upper limit of the therapeutic interval for warfarin [11]. It is also consistent with the generally accepted upper limit of INR for extractions in the region where the study was conducted.

The various groups of patients in the study were comparable. Today, atrial fibrillation is seldom an indication for warfarin, and DOACs are not indicated for patients with mechanical heart valves, which explain any notable discrepancies between these two groups [11].

The present study considered the occurrence of postoperative bleeding a crucial outcome measure. The findings suggest that postoperative bleeding appears to be less common for patients receiving DOACs compared to those receiving warfarin. Furthermore, in both groups, the patients were able to handle most bleeding themselves. These findings are consistent with previous studies [12–15] and underscore the importance of providing patients with comprehensive guidance on the management of minor bleeding. There are several possible explanations for why DOACs may be associated with a lower risk of bleeding than warfarin. DOACs have more predictable pharmacokinetics and pharmacodynamics with a shorter half-life, wider therapeutic interval, and fewer drug and food interactions, all of which may contribute to a more consistent anticoagulant effect and reduced need for monitoring. Another explanation may be that, unlike warfarin, DOACs do not interfere with tissue factor and factor VII [10].

Postoperative bleeding also occurs in patients who are not receiving anticoagulants. The prospective investigation of Rademacher et al. examined the risk of postoperative bleeding following the removal of third molars in young, healthy patients who had not taken any medication that could potentially affect haemostasis within the 10 days before treatment [16]. The authors indicated that the risk of any type of postoperative bleeding was 32%, a figure that is comparable to the risk observed in patients receiving DOACs in the present study (27%). Additionally, the risk of grade 2 postoperative bleeding appeared to be lower for the patients in the study by Rademacher et al. (1.4%) compared to the DOAC group in the present study (3.2%).

Some aspects of the subgroup analyses merit further discussion. The therapeutic interval for warfarin is typically within the range of 2.0–3.0. However, some of our study patients had an INR of 3.1. This may reflect reality. It is reasonable to assume that, occasionally, in general dental practice, extractions are also done when the INR of the patient is marginally above the desired range. Moreover, INR values should not be considered fixed [17]. In the present study, DOACs were grouped into higher- and lower-dose categories, like in the Brennan et al. study [14]. Clinically, it was deemed valuable to include the two patients who continuously took a lower dose of DOACs than recommended, given widespread acknowledgement that not all patients adhere to the full dosage of their prescribed medications [18]. In patients with moderate to severe renal impairment (eGFR 31–44 mL/min/1.73 m^2^), no instances of bleeding that necessitated assistance from dentists or other healthcare professionals occurred. It thus seems reasonable to assume that the benefit of supplementary renal function controls before extractions is limited.

Some variables had a significant impact on the outcomes. The duration of surgery predicted several outcomes and may be regarded as an indicator of the complexity of the surgical procedure. Furthermore, our findings suggest that an increase in systolic blood pressure is associated with a significantly elevated risk of postoperative bleeding. This lends further support to the generally accepted view that maintaining optimal blood pressure is important [19, 20]. Patients with elevated blood pressure were asked to contact their general practitioner.

In light of the findings of this study, some suggestions can be made for the management of patients receiving DOACs in clinical practice. The first recommendation is that patients continue to take their DOACs, without interruption, throughout simple *and* surgical tooth extractions. This approach is recommended from an ethical perspective, emphasising patient safety as a primary concern. Adherence to this recommendation ensures that the potential for increased risk of thromboembolic events, which is regarded as far more serious than the potential for increased incidence of local postoperative bleeding, is avoided. To mitigate the risk of bleeding, a further recommendation is to use the approach outlined in the Methods section. It seems likely that the referral departments in this study were primarily consulted for complex cases, which may account for a greater incidence of complications. Thus, the prevalence of bleeding and other complications may be lower in the general population compared to what we observed in this study.

Clear communication is important when implementing new recommendations. Clinician compliance can be enhanced by discussing patient safety priorities and the time saved by not having to discuss anticoagulant discontinuation with the patient’s general practitioner. Patient adherence to normal anticoagulant therapy during extractions is crucial and can be improved by informing patients of the potential risk of thromboembolic events associated with anticoagulant discontinuation and by providing clear instructions on how to manage postoperative bleeding at home.

Our study has its strengths and limitations. The study was a prospective, multicentre investigation, in which all eligible patients were invited to participate. Results were adjusted for several confounding variables, and specific variables that influenced the results were identified. Concerning limitations, performance bias may have influenced the results since the study was not conducted in a blinded manner. Second, since a randomised study was not feasible, some residual confounding may have occurred, despite the comprehensive characterisation of the DOAC and warfarin groups and the control of numerous variables. Third, as the study reported on multiple outcomes, inflation of the type I error rate may be possible. Conversely, however, other studies corroborate our findings [12–14]. Additionally, this report does not include any information regarding possible bleeding or other complications that may have occurred after the first seven postoperative days. Furthermore, some data, particularly on grade 1 postoperative bleeding, were based on patient-reported information which carries a risk of underreporting and recall bias. However, most patients were contacted soon after surgery and carefully questioned about any complications, which minimised these risks. Moreover, the observed difference between groups in postoperative bleeding may be greater than reported, due to the significantly higher proportion of patients in the warfarin group, compared with the DOAC group, who received resorbable local haemostatic agents in all extraction sockets *(p* = 0.03). However, the usefulness of this variable is limited by the lack of data on the specific local haemostatic agent used for each patient. Last, the final sample size for the warfarin group was below the required number calculated in the power analysis. For many patient groups, warfarin was replaced by DOACs during the study period, and this presented difficulties in recruiting patients to the warfarin group. Given the limited sample size, meta-analyses of comparable studies may be advisable to explore discrepancies between the DOAC and warfarin groups.

## Conclusions

The findings of this study indicate that patients taking DOACs without interruption may have a lower risk of bleeding compared to those on therapeutic levels of warfarin during simple and surgical tooth removal. To lower the risk of thromboembolic events associated with the cessation of anticoagulants, it is suggested that patients continue their DOACs without interruption throughout both routine and surgical tooth extractions, provided that local haemostatic measures are applied.

## Electronic supplementary material

Below is the link to the electronic supplementary material.

Additional file 1: Title and description of data: Complete output from the regression analyses

## Data Availability

The data that support the findings of this study are not publicly accessible in order to protect the anonymity of the research participants. The data are stored in a controlled-access data repository at Malmö University and are available from the corresponding author upon reasonable request.

## Abbreviations

DOAC: Direct oral anticoagulant
INR: International normalised ratio
eGFR: Estimated glomerular filtration rate
VAS: Visual analogue scale
OR: Odds ratio
CI: Confidence interval
IQR: Interquartile range
mmHg: Millimetres of mercury

## References

[1] Darwish G. The effect of direct oral anticoagulant therapy (DOACs) on oral surgical procedures: a systematic review. BMC Oral Health. 2023;23(1):743. 10.1186/s12903-023-03427-8. 37821865 10.1186/s12903-023-03427-8 PMC10566068

[2] Steffel J, Collins R, Antz M, Cornu P, Desteghe L, Haeusler KG, et al. 2021 European heart rhythm association practical guide on the use of Non-Vitamin K antagonist oral anticoagulants in patients with atrial fibrillation. Europace. 2021;23(10):1612–76. 10.1093/europace/euab065. 33895845 10.1093/europace/euab065 PMC11636576

[3] Douketis JD, Spyropoulos AC, Murad MH, Arcelus JI, Dager WE, Dunn AS, et al. Perioperative management of antithrombotic therapy: an American college of chest physicians clinical practice guideline. Chest. 2022;162(5):e207–43. 10.1016/j.chest.2022.07.025. 35964704 10.1016/j.chest.2022.07.025

[4] Ruff CT, Giugliano RP, Braunwald E, Hoffman EB, Deenadayalu N, Ezekowitz MD, et al. Comparison of the efficacy and safety of new oral anticoagulants with warfarin in patients with atrial fibrillation: a meta-analysis of randomised trials. Lancet. 2014;383(9921):955–62. 10.1016/S0140-6736(13)62343-0. 24315724 10.1016/S0140-6736(13)62343-0

[5] Johansson K, Gotrick B, Holst J, Tranaeus S, Naimi-Akbar A. Impact of direct oral anticoagulants on bleeding tendency and postoperative complications in oral surgery: a systematic review of controlled studies. Oral Surg Oral Med Oral Pathol Oral Radiol. 2023;135(3):333–46. 10.1016/j.oooo.2022.07.003. 36100547 10.1016/j.oooo.2022.07.003

[6] Mehran R, Rao SV, Bhatt DL, Gibson CM, Caixeta A, Eikelboom J, et al. Standardized bleeding definitions for cardiovascular clinical trials: a consensus report from the bleeding academic research consortium. Circulation. 2011;123(23):2736–47. 10.1161/CIRCULATIONAHA.110.009449. 21670242 10.1161/CIRCULATIONAHA.110.009449

[7] Johansson K, Lindstrom M, Alhabshi M, Ahmad M, Svensson PJ, Becktor JP. Estimation of blood loss in oral and maxillofacial surgery by measurements of low haemoglobin levels in mixtures of blood, saliva and saline: a laboratory study. J Oral Maxillofac Res. 2021;12(2):e3. 10.5037/jomr.2021.12203. 34377380 10.5037/jomr.2021.12203 PMC8326882

[8] Svensson R, Hallmer F, Englesson CS, Svensson PJ, Becktor JP. Treatment with local hemostatic agents and primary closure after tooth extraction in warfarin treated patients. Swed Dent J. 2013;37(2):71–7. https://www.ncbi.nlm.nih.gov/pubmed/23957141. 23957141

[9] Akaike H. Data analysis by statistical models. No Hattatsu. 1992;24(2):127–33. https://www.ncbi.nlm.nih.gov/pubmed/1567644. 1567644

[10] Hindley B, Lip GYH, McCloskey AP, Penson PE. Pharmacokinetics and pharmacodynamics of direct oral anticoagulants. Expert Opin Drug Metab Toxicol. 2023;19(12):911–23. 10.1080/17425255.2023.2287472. 37991392 10.1080/17425255.2023.2287472

[11] Joglar JA, Chung MK, Armbruster AL, Benjamin EJ, Chyou JY, Cronin EM, et al. 2023 ACC/AHA/ACCP/HRS guideline for the diagnosis and management of atrial fibrillation: A report of the American college of cardiology/american heart association joint committee on clinical practice guidelines. Circulation. 2024;149(1):e1–156. 10.1161/CIR.0000000000001193. 38033089 10.1161/CIR.0000000000001193 PMC11095842

[12] Andrade MVS, Andrade LAP, Bispo AF, Freitas LD, Andrade MQS, Feitosa OS, et al. Evaluation of the bleeding intensity of patients anticoagulated with warfarin or Dabigatran undergoing dental procedures. Arquivos Brasileiros De Cardiologia. 2018;111(3):394–9. 10.5935/abc.20180137. 30088558 10.5935/abc.20180137 PMC6173350

[13] Berton F, Costantinides F, Rizzo R, Franco A, Contarin J, Stacchi C, et al. Should we fear direct oral anticoagulants more than vitamin K antagonists in simple single tooth extraction? A prospective comparative study. Clin Oral Investig. 2019;23(8):3183–92. 10.1007/s00784-018-2739-9. 30392079 10.1007/s00784-018-2739-9

[14] Brennan Y, Gu Y, Schifter M, Crowther H, Favaloro EJ, Curnow J. Dental extractions on direct oral anticoagulants vs. warfarin: the DENTST study. Res Pract Thromb Haemostasis. 2020;4(2):278–84. 10.1002/rth2.12307. 32110759 10.1002/rth2.12307 PMC7040537

[15] Buchbender M, Schlee N, Kesting MR, Grimm J, Fehlhofer J, Rau A. A prospective comparative study to assess the risk of postoperative bleeding after dental surgery while on medication with direct oral anticoagulants, antiplatelet agents, or vitamin K antagonists. BMC Oral Health. 2021;21(1):504. 10.1186/s12903-021-01868-7. 34620135 10.1186/s12903-021-01868-7 PMC8499467

[16] Rademacher WMH, Rooijers W, Broekman MW, van der Slik BJ, van Diermen DE, Rozema FR. Nabloedingsrisico Na verwijdering Derde Molaar Bij Gezonde patiënten; Een prospectief observationeel klinisch Onderzoek [Postoperative risk of bleeding after removal of third molars in healthy patients - a prospective observational clinical study]. Ned Tijdschr Tandheelkd. 2024;131(7–08):307–15. 10.5177/ntvt.2024.07/08.24028. 38973659 10.5177/ntvt.2024.07/08.24028

[17] Bonar R, Favaloro EJ. Explaining and reducing the variation in inter-laboratory reported values for international normalised ratio. Thromb Res. 2017;150:22–9. 10.1016/j.thromres.2016.12.007. 27998809 10.1016/j.thromres.2016.12.007

[18] Glasziou P, Straus S, Brownlee S, Trevena L, Dans L, Guyatt G, et al. Evidence for underuse of effective medical services around the world. Lancet. 2017;390(10090):169–77. 10.1016/S0140-6736(16)30946-1. 28077232 10.1016/S0140-6736(16)30946-1

[19] Ndrepepa G, Groha P, Lahmann AL, Lohaus R, Cassese S, Schulz-Schupke S, et al. Increased bleeding risk during percutaneous coronary interventions by arterial hypertension. Catheter Cardiovasc Interv. 2016;88(2):184–90. 10.1002/ccd.26272. 26526702 10.1002/ccd.26272

[20] Raven PB, Chapleau MW. Blood pressure regulation XI: overview and future research directions. Eur J Appl Physiol. 2014;114(3):579–86. 10.1007/s00421-014-2823-z. 24463603 10.1007/s00421-014-2823-z PMC3955090

