# Supplementary material for: Continuous use of direct oral anticoagulants during and after simple and surgical tooth extractions: a prospective clinical cohort study
Source: BMC Oral Health. 2025 Apr 12;25:554. doi: 10.1186/s12903-025-05949-9 (PMC11993950; doi:10.1186/s12903-025-05949-9)
Supplement: Supplementary file 1 — Additional file 1: Title and description of data: Complete output from the regression analyses [file 12903_2025_5949_MOESM1_ESM.docx]

**Additional file 1** Complete output from the regression analyses

Outcome: Postoperative bleeding, any type

| **Predictor** | **Odds ratio**  **(95% confidence interval)** | ***p*** |
| --- | --- | --- |
| *Anticoagulant* |  |  |
| Warfarin | 1 |  |
| Direct oral anticoagulants | 0.66 (0.28 – 1.58) | 0.35 |
| *Characteristics* |  |  |
| Extraction from upper jaw | 1.75 (0.76 – 4.03) | 0.19 |
| Surgical extraction | 1.01 (0.35 – 2.92) | 0.99 |
| Extraction of molar | 1.53 (0.60 – 3.91) | 0.38 |
| Extraction of ˃ one tooth | 3.33 (1.43 – 7.76) | 0.01 |
| Diabetes | 0.48 (0.17 – 1.36) | 0.17 |
| Diastolic blood pressure | 0.98 (0.94 – 1.01) | 0.15 |
| Systolic blood pressure | 1.02 (1.00 – 1.04) | 0.04 |
| *Country of birth* |  |  |
| Sweden | 1 (reference) |  |
| Europe, except for Sweden | 1.86 (0.52 – 6.63) | 0.34 |
| Non-European | 7.65 (1.52 – 38.41) | 0.01 |
| Unknown | 4.80 (0.85 – 26.99) | 0.08 |
| *Sex* |  |  |
| Male | 1 |  |
| Female | 1.03 (0.46 – 2.29) | 1.00 |
| *Level of education* |  |  |
| Lower secondary or lower | 1 (reference) |  |
| Upper secondary | 0.93 (0.34 – 2.58) | 0.90 |
| Post-secondary or higher | 1.85 (0.72 – 4.79) | 0.20 |
| Unknown | 0.27 (0.03 – 2.66) | 0.26 |
| *Treatment institution* |  |  |
| Malmö University | 1 (reference) |  |
| Public Dental Service | 1.53 (0.49 – 4.81) | 0.47 |
| Skåne University Hospital | 0.59 (0.23 – 1.46) | 0.25 |
| *Age* |  |  |
| Below 65 | 1 (reference) |  |
| 65 – 79 | 1.22 (0.36 – 4.15) | 0.76 |
| 80 and above | 1.01 (0.28 – 3.58) | 0.99 |

Outcome: Postoperative bleeding, Grade 2

| **Predictor** | **Odds ratio**  **(95% confidence interval)** | ***p*** |
| --- | --- | --- |
| *Anticoagulant* |  |  |
| Warfarin | 1 |  |
| Direct oral anticoagulants | 0.30 (0.08 – 1.05) | 0.06 |
| *Characteristics* |  |  |
| Extraction from upper jaw | 2.63 (0.62 – 11.15) | 0.19 |
| Surgical extraction | 3.80 0.48 – 32.78) | 0.20 |
| Diastolic blood pressure | 1.00 (0.95 – 1.05) | 0.90 |
| Extraction of ˃ one tooth | 1.16 (0.31 – 4.43) | 0.82 |

Outcome: Complications other than postoperative bleeding

| **Predictor** | **Coefficient**  **(95% confidence interval)** | ***p*** |
| --- | --- | --- |
| *Anticoagulant* |  |  |
| Warfarin | 1 |  |
| Direct oral anticoagulants | 1.18 (0.23 – 5.91) | 0.84 |
| *Characteristics* |  |  |
| Extraction from upper jaw | 0.22 (0.04 – 1.10) | 0.06 |
| Diastolic blood pressure | 1.06 (0.99 – 1.12) | 0.07 |

Outcome: Perioperative bleeding volume (mL)

| **Predictor** | **Coefficient**  **(95% confidence interval)** | ***p*** |
| --- | --- | --- |
| *Anticoagulant* |  |  |
| Warfarin | 0 |  |
| Direct oral anticoagulants | 0.36 (-3.02 – 3.75) | 0.83 |
| *Characteristics* |  |  |
| Extraction from upper jaw | -3.35 (-6.66 – -0.03) | 0.05 |
| Surgical extraction | 7.03 (3.37 – 10.69) | 0.00 |
| Extraction of molar | 4.37 (0.74 – 8.00) | 0.02 |
| Extraction of ˃ one tooth | 2.99 (-1.65 – 7.62) | 0.21 |
| Diabetes | -0.77 (-4.40 – 2.86) | 0.68 |
| Diastolic blood pressure | 0.11 (-0.02 – 0.24) | 0.09 |
| Systolic blood pressure | 0.01 (-0.06 – 0.09) | 0.70 |
| Local anasesthesia/tooth (mL) | -0.10 (-1.39 – 1.19) | 0.89 |
| *Country of birth* |  |  |
| Sweden | 0 (reference) |  |
| Europe, except for Sweden | 0.15 (-4.49 – 4.80) | 0.95 |
| Non-European | 9.78 (2.40 – 17.17) | 0.01 |
| Unknown | -1.70 (-10.46 – 7.06) | 0.70 |
| *Sex* |  |  |
| Male | 0 |  |
| Female | -0.78 (-3.78 – 2.23) | 0.61 |
| *Level of education* |  |  |
| Lower secondary or lower | 0 (reference) |  |
| Upper secondary | -0.79 (-4.63 – 3.04) | 0.68 |
| Post-secondary or higher | -0.38 (-4.08 – 3.31) | 0.84 |
| Unknown | -1.13 (-11.01 – 8.74) | 0.82 |
| *Treatment institution* |  |  |
| Malmö University | 0 (reference) |  |
| Public Dental Service | -1.94 (-7.25 – 3.38) | 0.47 |
| Skåne University Hospital | 3.92 (0.41 – 7.43) | 0.03 |
| *Age* |  |  |
| Below 65 | 0 (reference) |  |
| 65 – 79 | -1.48 (-5.84 – 2.87) | 0.50 |
| 80 and above | -1.99 (-6.85 – 2.87) | 0.42 |

Outcome: Surgeon assessment of the impact of bleeding on procedure complexity

| **Predictor** | **Coefficient**  **(95% confidence interval)** | ***p*** |
| --- | --- | --- |
| *Anticoagulant* |  |  |
| Warfarin | 0 |  |
| Direct oral anticoagulants | 0.16 (-0.12 – 0.45) | 0.27 |
| *Characteristics* |  |  |
| Extraction from upper jaw | -0.00 (-0.29 – 0.28) | 0.98 |
| Surgical extraction | 0.40 (0.09 – 0.71) | 0.01 |
| Extraction of molar | 0.08 (-0.23 – 0.39) | 0.61 |
| Extraction of ˃ one tooth | 0.13 (-0.26 – 0.53) | 0.50 |
| Diabetes | 0.14 (-0.19 – 0.45) | 0.38 |
| Diastolic blood pressure | 0.00 (-0.01 – 0.02) | 0.40 |
| Systolic blood pressure | 0.00 (-0.00 – 0.01) | 0.33 |
| Local anesthesia/tooth (mL) | 0.02 (-0.09 – 0.13) | 0.71 |
| *Country of birth* |  |  |
| Sweden | 0 (reference) |  |
| Europe, except for Sweden | -0.16 (-0.56 – 0.24) | 0.44 |
| Non-European | 0.36 (-0.28 – 0.99) | 0.27 |
| Unknown | -0.17 (-0.83 – 0.48) | 0.61 |
| *Sex* |  |  |
| Male | 0 |  |
| Female | -0.20 (-0.46 – 0.05) | 0.12 |
| *Level of education* |  |  |
| Lower secondary or lower | 0 (reference) |  |
| Upper secondary | -0.34 (-0.67 – -0.02) | 0.04 |
| Post-secondary or higher | -0.33 (-0.65 – -0.01) | 0.04 |
| Unknown | -0.22 (-0.99 – 0.55) | 0.57 |
| *Treatment clinic* |  |  |
| Malmö University | 0 (reference) |  |
| Public Dental Service | -0.36 (-0.81 – 0.09) | 0.11 |
| Skåne University Hospital | 0.05 (-0.25 – 0.34) | 0.76 |
| *Age* |  |  |
| Below 65 | 0 (reference) |  |
| 65 – 79 | -0.47 (-0.84 – -0.10) | 0.01 |
| 80 and above | -0.38 (-0.79 – 0.02) | 0.07 |
